# Discrepancy in clinical versus radiological parameters describing deformity due to brace treatment for moderate idiopathic scoliosis

**DOI:** 10.1186/1748-7161-2-18

**Published:** 2007-12-03

**Authors:** Tomasz Kotwicki, Edyta Kinel, Wanda Stryla, Andrzej Szulc

**Affiliations:** 1 Department of Pediatric Orthopedics and Traumatology, University of Medical Sciences of Poznan, ul. 28 Czerwca 1956 roku nr 135; 61-545 Poznan, Poland; 2 Department of Rehabilitation, University of Medical Sciences of Poznan, ul. 28 Czerwca 1956 roku nr 135; 61-545 Poznan, Poland

## Abstract

**Background:**

The shape of the torso in patients with idiopathic scoliosis is considered to reflect the shape of the vertebral column, however the direct correlation between parameters describing clinical deformity and those characterizing radiological curvature was reported to be weak. It is not clear if the management proposed for scoliosis (physiotherapy, brace, surgery) affects equally the shape of the axial skeleton and the surface of the body. The aim of the study was to compare clinical deformity of (1) idiopathic scoliosis girls being under brace treatment for radiological curves of 25 to 40 degrees and (2) non treated scoliotic girls matched for age and Cobb angle.

**Methods:**

Cross-sectional study of 24 girls wearing the brace versus 26 girls without brace treatment, matched for age and Cobb angle. Hypothesis: Patients wearing the brace for more than 6 months, when comparing to patients without brace, may present different external morphology of the trunk, in spite of having similar Cobb angle. Material. Inclusion criteria: girls, idiopathic scoliosis, growing age (10–16 years), Cobb angle minimum 25°, maximum 40°. The braced group consisted of girls wearing a TLSO brace (Cheneau) for more than 6 months with minimum of 16 hours per day. The non-braced group consisted of girls first seen for their spinal deformity, previously not treated. The groups presented similar curve pattern. Methods. Scoliometer exam: angle of trunk rotation at three levels of the spine: upper thoracic, main thoracic, lumbar or thoracolumbar. The maximal angle was noted at each level and the sum of three levels was calculated. Posterior trunk symmetry index (POTSI) and Hump Sum were measured using surface topography.

**Results:**

Cobb angle was 34.9° ± 4.8° in braced and 32.7° ± 4.9° in un-braced patients (difference not significant). The age was 14.1 ± 1.6 years in braced patients and 13.1 ± 1.9 years in un-braced group (p = 0.046). The value of angle of trunk rotation in the main curvature was 8.4° ± 2.7°in braced and 11.4° ± 2.7° in un-braced patients (difference extremely significant, p = 0.0003). The value of the sum of angles of trunk rotation at three levels of the trunk was 12.8° ± 4.6° in braced and 16.5° ± 3.8° in un-braced patients (difference very significant, p = 0.0038). The POTSI did not differ significantly between the groups (p = 0.78), the Hump Sum values were not quite different (p = 0.07).

**Conclusion:**

(1) Adolescent girls wearing the brace for idiopathic scoliosis of 25 to 40 degrees of Cobb angle, reveal smaller clinical rotational deformity of their back than non-treated girls having similar radiological deformity. (2) Evaluation of the results of treatment for idiopathic scoliosis should consider parameters describing both clinical and radiological deformity.

## Background

Brace treatment is a standard management for progressive idiopathic scoliosis of moderate Cobb angle; it is usually recommended for angles of 25 to 40 degrees and, if residual growth of the spine is expected [1]. Clinical assessment as well as radiological measurements are the two basic examinations for evaluation of the deformity. The main clinical parameters are: the C7 plumb line, axillary plumb line, shoulder and hip asymmetry, which can be objectively measured with surface topography using the POTSI index [2], as well as the angle of trunk rotation, which is assessed with the scoliometer [3]. On the radiological exam the Cobb angle, apical vertebra translation, angle of vertebral axial rotation are usually used, however the Cobb angle is considered the most universal parameter to evaluate the curve magnitude [4]. It seems logical and is generally admitted that there exist some parallelism between the degree of intensity of clinical and of radiological parameters describing the deformity. The more severe the curve in terms of Cobb angle the more the surface deformity is pronounced.

However in practice one can often notice discrepancy between results of both exams. The efforts of the researchers who were seeking for a clinical parameter (including surface topography measures), which would perfectly correlate with the Cobb angle have failed, probably because the Cobb angle signifies just the tilt of the two end vertebrae of the curve, projected on the surface parallel to the frontal plane of the body. According to Bunnell, "although there is a significant correlation between clinical deformity and radiological measurement, the standard deviation is high" [5]. Age is a factor that influence the correlation between the surface and the spinal deformity. Grivas et al. reported a weak correlation in younger children, and a stronger one in older children [6].

**Figure 1:**
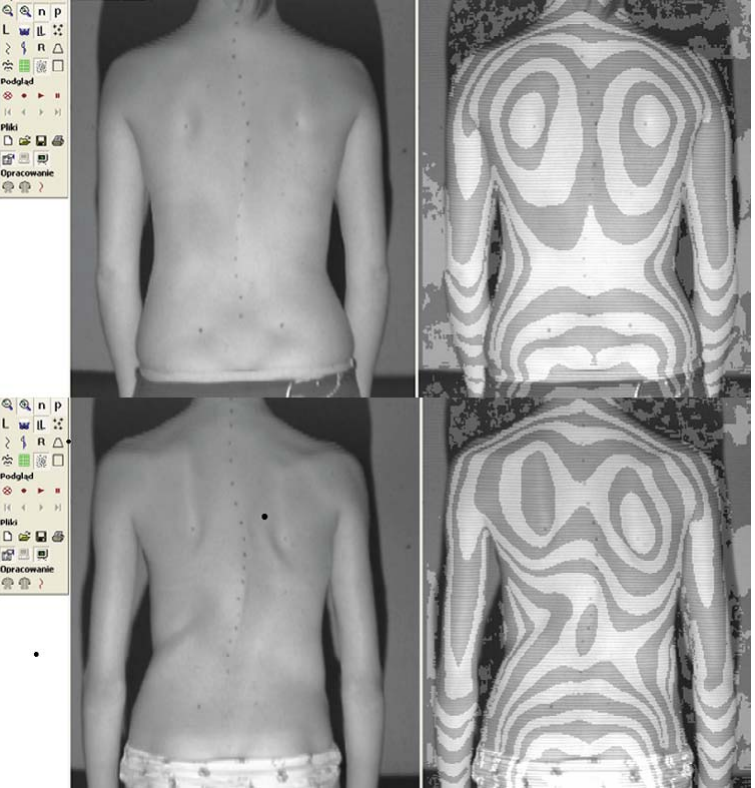
Two girls with 40 degrees Cobb angle right thoracic scoliosis each. Photo (left) and surface topography image (right) of the back. The raster stereography image is presented in pseudo-Moire form for convenience. **Top**. 14-year-old girl, has been wearing Cheneau brace for 2.5 years, Cobb = 39°, Bunnell angle of trunk rotation of the main curvature = 8°, Risser 4. **Bottom**. 13-year-old girl, previously non treated, Cobb = 40°, Bunnell angle of trunk rotation of the main curve = 15°, Risser 1.

In this study we aimed to verify if the treatment of scoliosis with a brace can influence the relation of the clinical versus radiological image of the trunk. The aim of the study was to compare the clinical deformity in two groups of patients presenting similar radiological deformity: the first treated with a corrective brace and the second non treated. The hypothesis was that girls treated for idiopathic scoliosis with a brace for a period longer than 6 months, having the curves of 25 to 40 degrees of Cobb angle, may present significantly different morphology of the trunk, comparing to girls matched for age and Cobb angle but not treated (Figure 1).

## Methods

**Figure 2:**
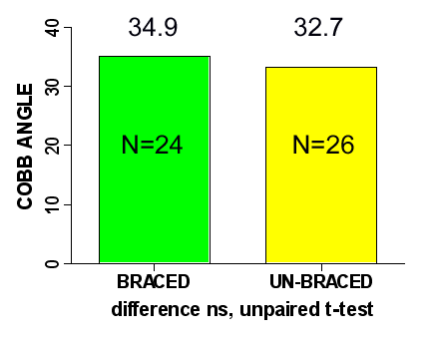
Cobb angle value in the brace-treated and in the non braced group.

Inclusion criteria were as follows: girls, idiopathic scoliosis, growing age (10–16 years), Cobb angle of minimum 25° and maximum 40°. Fifty consecutive girls were included in the study and distributed into two groups. The braced group consisted of 24 girls wearing a TLSO (Cheneau brace) for more than 6 months with a minimum time of wearing of 16 hours per day. The non-braced group consisted of 26 girls first seen for their spinal deformity, previously not treated. The age of braced group was 14.1 ± 1.6 years and the age of non braced group was 13.1 ± 1.9 years, difference slightly significant (unpaired t test, p = 0.046). The Cobb angle was 34.9° ± 4.8° (from 25° to 40°) and 32.7° ± 4.9° (from 25° to 40°) respectively, difference not significant (unpaired t-test, p > 0.05), Figure 2. Risser sign value was less than 3 in 12 girls from the braced group and in 23 girls of the non-braced group. The curve pattern was similar in both groups (Table 1). In the non-braced group (N = 26), there was 15 girls with single curvatures (10 thoracic and 5 thoracolumbar) and 11 girls with double curvatures (right thoracic and left lumbar). In the braced group (N = 24), there was 18 girls with single curvatures (11 thoracic and 7 thoracolumbar) and 6 girls with double curvatures (right thoracic and left lumbar). The proportion of patients with single to those with double curvatures was similar in both groups (Fisher's exact test). Also the proportion of thoracic to thoracolumbar to lumbar curvatures was not significantly different between the two groups (p > 0.05). The braces were all made in the same workshop and the treatment was managed by the same physician (T.K.).

**Table 1:** Curve pattern in both groups. There was no significant difference between groups, concerning the proportion of single to double curves nor the proportion of thoracic to thoracolumbar to double curves

Curve type	Lenke type	Braced group	Non braced group
Thoracic	I	11	10
Thoracolumbar	V	7	5
Double	III	6	11
All		24	26

The Cobb angle in patients treated with a brace was assessed on a radiograph made out of brace. The patients received an X-ray request during the previous visit; the day of the current visit they slept in brace, they took it out in the morning, and came with the brace in hands for consultation. The patients stayed out of brace for an average period of six hours, with a minimum of two hours, before making the spinal radiograph. The patient presented at the physician with her current radiograph, then the clinical parameters were checked and the surface topography exam was performed.

The following clinical parameters were considered: C7 plumb line, left and right axillary plumb line symmetry, angle of trunk rotation (ATR or Bunnell angle) measured with the scoliometer of Bunnell [3]. The pelvis was level in all patients and none of the patients needed a lift to correct the pelvis tilt. Standing forward bending position was used to measure the trunk rotation. All scoliometer measurements were done by the same observer (T.K.), who previously checked the rates of inter-observer and intra-observer reliability, and obtained a high intra-observer agreement [7]. The child was standing symmetrically on both feet, knees in extension, feet set at the width of the hips; she executed a gradual slow forward flexion, which was stopped at the physician's command, at the moment that the part of the trunk to be examined was positioned horizontally. The scoliometer was gently placed on the skin without pressing down, transversally to the long spinal axis, with the central notch of the scoliometer over the spinous process and the angle of trunk rotation was noted in degrees. The ATR was measured at three levels of the spine (proximal thoracic, main thoracic and thoracolumbar or lumbar) and the sum of three ATRs was calculated. In the brace treated group the value of the angle of trunk rotation of the main curve was found in the charts, and compared to the current ATR value. Surface topography examination was performed the same day as clinical and radiological examination. Raster stereography was used (CQ Electronic, Wroclaw, Poland). The POTSI index was calculated for the frontal plane assessment and the Hump Sum (HS) for the transverse plane assessment. The HS was composed of maximum rotation at three levels of the spine (proximal thoracic, main thoracic and thoracolumbar or lumbar). Kolmogorov-Smirnof test was used to check normality and Fisher-Snedecor test to check equality of standard deviations between groups. Unpaired t-test was used to compare means, p value of 0.05 considered significant.

## Results

**Figure 3:**
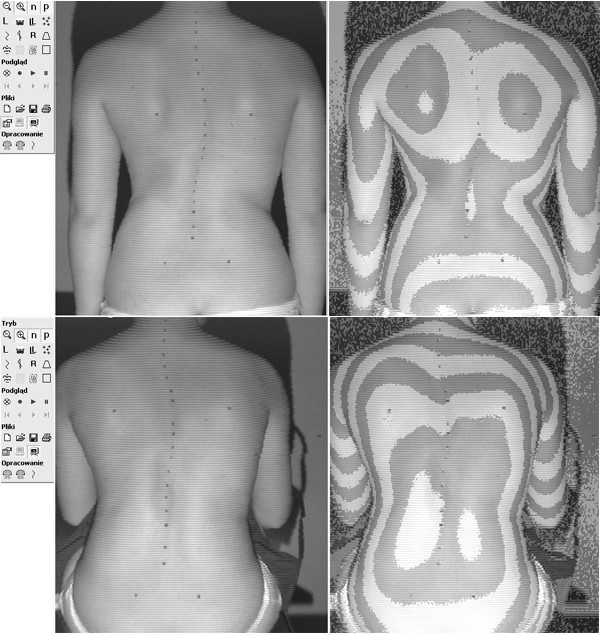
14 years and 5 months old girl, treated with Cheneau brace for 2.5 years, including 1.5 years full time and 1.0 year part time wearing. Thoracic Cobb angle 36°, lumbar Cobb angle 32°, Risser sign 4. Two years after menarche. Main curve ATR = 7°, sum of three ATRs = 13°, POTSI = 25.5, HS = 26. Raster stereography image in standing (top) and sitting forward bending (bottom) position. There is less clinical rotational deformity, comparing to the non-treated girl presented in the Figure 5, in spite of more pronounced radiological deformity, illustrated in the Figure 4, comparing to the radiological deformity of the non-treated girl, presented in the Figure 6.

**Figure 4:**
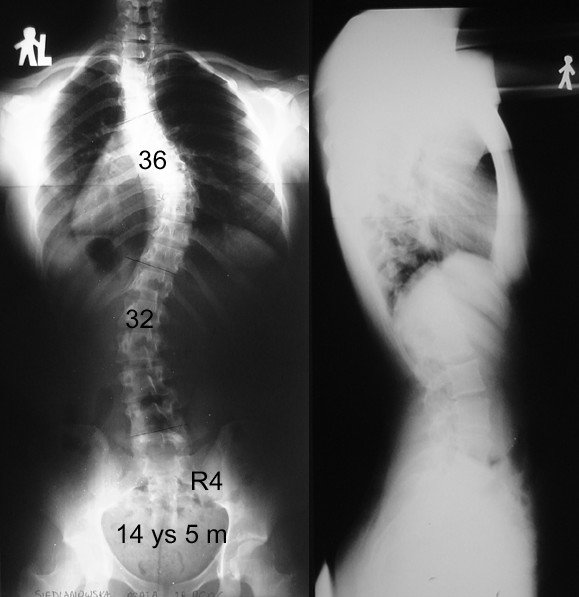
Standing AP and lateral radiographs of the patient from the Figure 3, brace-treated group.

**Figure 5:**
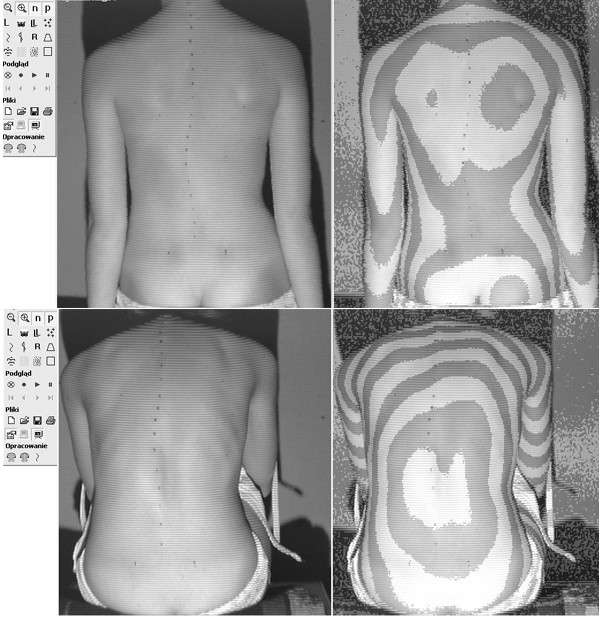
12 years and 10 months old girl, first seen for scoliosis. Thoracic Cobb angle 22°, lumbar Cobb angle 25°, Risser sign 0. Two months after menarche. Main curve ATR = 10°, sum of three ATRs = 20°, POTSI = 25.2, HS = 14. Raster stereography image in standing (top) and sitting forward bending (bottom) position.

**Figure 6:**
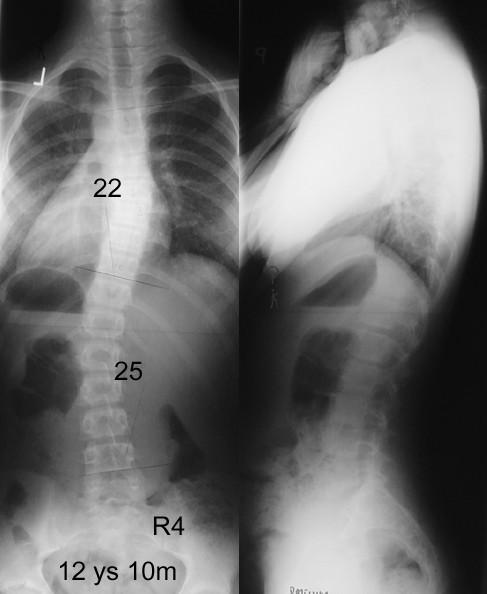
Standing AP and lateral radiographs of the patient from the Figure 5, non-treated group.

In spite of similar Cobb angle the clinical parameters revealed important discrepancy between the braced and non braced patients, demonstrating less clinical deformity in the braced group (Table 2.). This was found especially for the trunk rotation (ATR main curve and ATR three levels), which revealed very significant differences between groups, but not for the frontal plane assessment. The POTSI value did not differ between groups (p = 0.78, unpaired t-test with Welch correction) as well as C7 plumb line and axillary plumb line did. The correlation between the primary curve Cobb angle and primary curve Bunnell angle was r = 0.36 (p < 0.05) in the non braced patients, significantly higher than in the braced group, r = 0.22 (p < 0.05). There was a higher correlation between the sum of ATRs at three levels (in forward flexion) and the standing Hump Sum in the braced group (r = 0.42, p < 0.05) than in the non braced group (r = 0.23, p < 0.05). In the brace treated patients the mean value of the ATR of the main curvature was significantly lower, than the value registered before starting the treatment (8.4 ± 2.7° versus the initial value of 10.2 ± 2.9°, p = 0.0025, paired t test). Examples of patients from both groups are presented in Figures, as follows: clinical image of a brace-treated girl in Figure 3, radiological examination of this girl in Figure 4, clinical image of a non-treated girl in Figure 5, radiological examination of this girl in Figure 6.

**Table 2:** Values of the clinical parameters in braced patients versus non braced patients matched for the Cobb angle. The mean and the standard deviation are presented. HS – Hump Sum. S – difference significant. NS – difference not significant

Parameter	Brace-treated patients N = 24	Non-braced patients N = 26	Significance of difference	P value
Cobb angle	34.9° ± 4.8°	32.7° ± 4.9°	NS	0.1
ATR main curve	8.4° ± 2.7°	11.4° ± 2.7°	extremely S	0.0003
ATR three levels	13.2° ± 4.7°	16.2° ± 4.2°	very S	0.0038
HS standing	14.2° ± 4.6°	17.0° ± 5.9°	not quite S	0.07
C7 plumb line	1.1 ± 0.9 cm	1.0 ± 0.9 cm	NS	0.61
Axillary plumb line	1.9 ± 1.5 cm	2.2 ± 1.6 cm	NS	0.52
POTSI index	26.1 ± 18.3	24.9 ± 11.8	NS	0.78

## Discussion

This study points on the discrepancy between surface image of the trunk and radiologically assessed curvature (Cobb angle) in adolescent girls submitted to the treatment of progressive scoliosis with a corrective spinal orthosis. Taking into consideration the same gender, age, type of scoliosis and the Cobb angle – the differences in clinical parameters found in between groups should be attributed to the influence of orthosis on the trunk shape. Although the study is not a longitudinal study, we compared the values of the angle of trunk rotation before start of bracing and under brace treatment. We found a significant decrease of rotation in the main curvature (8.4 ± 2.7° versus 10.2 ± 2.9°). This finding is an additional argument that the brace was responsible for the lower values of the ATR in the braced group. In our study the rotation deformity, evaluated with the scoliometer (ATR) and with surface topography (HS) was diminished in the braced group. The rotation deformity in the transverse plane of the body remains the essential expression of idiopathic scoliosis. Stokes et al. reported that the measurement of the back surface asymmetry with surface topography that gave the highest correlation with the skeletal deformity was the axial rotation (back surface axial rotation versus vertebral axial rotation) [8]; the natural history patients being considered. Correlation coefficient of the ATR (clinical parameter) versus Cobb angle (radiological parameter) revealed lower values in the patients wearing the brace, which may reflect the increased discrepancy of surface versus skeletal deformity under brace treatment. In more severe structural scoliosis, usually with the Cobb angle greater than 50 degrees, the trunk rotation may spread out of the main curve proximally or distally. In our patients we did not find the situation, that the trunk rotation was oriented towards the same side at two adjacent levels (for example right main thoracic and right proximal thoracic). Therefore, to obtain the sum of rotation, we simply added the values read at three levels of the trunk: proximal thoracic, main thoracic and thoracolumbar/lumbar. There was no significant difference in parameters describing frontal plane asymmetry, namely the POTSI index, C7 plumb line and axillary plumb line. Usually, single curves cause more important clinical deformity than double curves. James stated that in double scoliosis "clinically the pattern is not very deforming for each of the structural curves balances the other" [9]. In this study the groups presented the same proportion of curve pattern: thoracic, thoracolumbar or double (thoracic and lumbar). There was a slightly higher proportion of single curvatures in the group managed with a brace, but not significant; a better clinical image of the back was noted in this group, in spite of such proportion. It was somewhat surprising, that both groups did not differ significantly according to the parameters describing frontal plane asymmetry (C7 plumb line, axillary plumb line, POTSI index). Possible reason is a small deformity in the non-braced group, the mean POTSI being within the normal range [10]. Another explication is, that it is the scoliotic curvature to straighten with brace, not the shoulders, scapulae, waists or other distant body parts. Voluntary imbalance of the shoulders or waist lines is sometimes introduced by the brace, in order to achieve better curve correction. The girls in the brace-treated group were slightly older (p = 0.046). According to Grivas et al. [6], they should present a better correlation between the Bunnell angle and the Cobb angle, than the non-treated group, which was not the case in our study (r = 0.22 versus r = 0.36). The interpretation of this finding is that the deformity in the treated children is corrected to a certain degree, due to applied conservative treatment using a brace.

Clinical to radiological discrepancy in idiopathic scoliosis was pointed out by James [9] who published the photos of the back of four girls with 70 degrees of curvature each, having largely different cosmetic appearance due to different curve location. The weak relationship of the rib prominence and Cobb angle was already reported by Thulbourne and Gillespie [11] however the influence of conservative treatment on further weakness of this relation has not been exploited. Ono [12] presented results of radiographic exam and surface topography in 504 patients with untreated idiopathic scoliosis and found the discrepancy between the Hump Sum and the Cobb angle. Grosso and Negrini [13] found no correlation between Cobb angle and clinical parameters (ATR, hump height, distance of the spinous process from the plumb line) in a cohort of 116 patients with moderate degree scoliosis. Goldberg et al. [14] identified significant but not complete correlation between Cobb angle and topography angle and supported surface topography as an adjunct to radiography. The same team developed better understanding of the fact that Cobb angle and surface parameters are not measuring the same aspect of the deformity, by proposing and testing new surface topography measures to quantify left-right asymmetry [15]. Grivas et al. indicated that the rib hump is not wholly a secondary effect, as the ribs themselves are asymmetric, and postulated that the deformity of the thorax develops first and this of the central axis succeeds [16].

In spite of an apparent consensus that Cobb angle cannot stand for surface deformity, the published results of brace treatment for progressive idiopathic scoliosis are most often based on the analysis of plane radiographs only, with special respect to the Cobb angle [17-19]. Coillard et al. [20], who previously proposed a valuable Freepoint system to evaluate relationship among various parts of the body, presented the results of the SpineCor brace limited to the Cobb angle analysis. Emans et al. [21] developed a detailed analysis of the results of the Boston bracing system, discussing the influence of various morphological parameters, such as curve type, curve apex location, vertebral axial rotation and in-brace initial correction on the final outcome however the radiological data were exclusively considered. Katz and Durrani [22] studied the curves of 36 to 45 degrees managed with the Boston brace to determine factors influencing the outcome but they limited the clinical data to gender, age, menarchial status, height, weight and brace wear schedule, avoiding any information on the shape of the patients' trunk.

One of the most important recent publications in the field is the SRS Committee report on standardization of criteria for adolescent idiopathic scoliosis brace studies [23]. The proposed criteria of outcome include: (1) Cobb angle progression, (2) Cobb angle exceeding 45°, (3) surgery recommended or undertaken. The authors analyzed 32 contributive brace studies but did not reported any clinical parameters assessing the brace effectiveness. In the analysis of "potentially useful additional variables" the SRS Committee enumerated curve pattern, curve magnitude, curve rotation, menarchial status, in-brace correction, skeletal maturation and peak height velocity. The parameters describing the shape of the body of children with idiopathic scoliosis were not considered.

The minority of authors reporting on bracing results for idiopathic scoliosis consider both clinical and radiological data. Rigo used Cobb and Perdriolle angles for radiographic evaluation while the lateral deviation, rotation, trunk imbalance, pelvis tilt and torsion were applied for surface evaluation [24]. The same author published a case report on a durable Cobb angle correction with a brace combined with Bunnell angle correction and surface topography lateral deviation correction [25]. Grivas et al. considered the Bunnell angle to assess the effect of Dynamic Derotation Brace [26]. The fundamental study of Nachemson and Peterson on the effectiveness of bracing included plumb line balance assessment, increase in height during the first year of observation and the presence and extent of a rib hump [27].

Few papers were found that discuss directly the question whether the management of scoliotic patients with a corrective orthosis can influence the difference in the clinical versus radiological outcome. Pham et al. reported on a series of 63 patients managed with Cheneau orthosis who presented significant reduction of the rib hump but not accompanied by a reduction of radiological rotation at the final follow-up 2 years after discontinuing the brace [28]. Weiss reported a case of radiological progression under brace treatment but combined with reduction of surface trunk rotation and surface lateral deviation [29].

Our study demonstrates that the phenomenon of discrepancy of clinical versus radiological measures should be considered by the physicians. The current inexplicable tendency seem to be to omit clinical data describing how the patient feels and how she looks like. Instead the ciphers read by the physician from the radiograph are supported. Nevertheless the improvement of the back shape is a recognized factor influencing the compliance.

From the patient's perspective it seems essential to realize that bracing is capable not only to stabilize radiological parameters but to improve cosmetic appearance. External image of the deformity have the impact on the general health perception, self-estimation as well as on emotional and social functioning. Clinical correction combined with the radiological stabilization appears as an attractive therapeutic option for patients with moderate curves which otherwise are known not to interfere with the patients' health and function throughout their life [30].

On the other hand we would like to stress that all patients in both groups presented radiologically progressive scoliosis, and at the same time these curves were at risk for further progression due to incomplete maturation. The therapy was not undertaken for cosmetic reasons and we do not have intention to recommend such an annoying management if the risk of progression is sufficiently low.

## Conclusion

1. Adolescent girls wearing the brace for idiopathic scoliosis of 25 to 40 degrees may reveal smaller clinical deformity than non-treated girls presenting similar radiological deformity.

2. Due to discrepancy between clinical and radiological outcome, the evaluation of the results of scoliosis treatment should take into consideration clinical parameters and not only radiological data.

## Competing interests

The author(s) declare that they have no competing interests.

## Authors' contributions

T.K. study design, data acquisition, data analysis and interpretation, manuscript drafting

E.K. data collection, analysis and interpretation

W.S. data interpretation, manuscript revision

A.S. data interpretation, manuscript revision

All authors read and approved the final manuscript
